# Early relapse of inflammatory breast carcinoma treated with lapatinib and capecitabine: Ten years of complete response

**DOI:** 10.1111/tbj.13606

**Published:** 2019-09-29

**Authors:** Roberto Martin Huertas, María Fernández Abad, Elena Corral de la Fuente, Juan José Serrano Domingo, Noelia Martínez Jáñez

**Affiliations:** ^1^ Medical Oncology Department Ramon y Cajal University Hospital Madrid Spain

Inflammatory breast carcinoma (IBC) is a rare form of breast cancer characterized by a rapid onset of diffuse skin erythema, edema, induration, and warmth of the breast. Biologically, IBC is usually hormone receptor (HR) negative and experience an overexpression of human epidermal growth factor receptor 2 (HER2), with a high mitotic index (MIB‐1 > 20%). Median overall survival is poor.^1^

Lapatinib is an oral drug that inhibits the tyrosine kinases of HER2 and epidermal growth factor receptor type 1 (EGFR) and has demonstrated clinical activity in trastuzumab‐refractory states.^2^ Lapatinib has also shown clinical activity in IBC, but evidence in this field is scarce.^3^, ^4^, ^5^, ^6^, ^7^, ^8^, ^9^

We present the case of an approximately ten‐year maintained complete response using lapatinib and capecitabine to treat an IBC early relapse after trastuzumab treatment.

A 34‐year‐old woman was diagnosed with multifocal invasive ductal carcinoma of the right breast (grade III). The immunohistochemical study showed the lesion tested negative for HR and positive for HER2 protein expression (3+). MIB‐1 expression was high, more than 25%, and a strong nuclear P53 expression was established. The patient was diagnosed with locally advanced breast cancer (cT2cN1M0).

In April 2007, neo‐adjuvant chemotherapy was initiated using doxorubicin plus cyclophosphamide during four cycles, followed by docetaxel administered concurrently with trastuzumab for an additional four cycles. Complete radiological and clinical responses were achieved. A breast‐conserving surgery was performed on the right breast in November 2007.

Final pathological results were consistent with multifocal invasive ductal carcinoma. The main focal lesion was 2.2 cm with a high grade (III), intraductal component in the main tumor was between 25% and 75%, and lymphovascular invasion was evident. Histological response was classified as no response or minimal response (Miller‐Payne grade 1). Clear margins were obtained, and no affected lymph nodes were noted among the 16 that were removed.

Trastuzumab was given every three weeks for a complete year. During this time, the patient also received radiotherapy by irradiation of the right breast volume and the regional lymph node area, as well as brachytherapy.

In January 2009, six months after completing trastuzumab therapy, the patient presented a local recurrence in the right breast. The recurrence was diagnosed as inflammatory carcinoma (dermal lymphatic invasion) with infiltrating ductal carcinoma histology (grade III, HR negative, HER2 positive, p53 positive, MIB‐1 > 40%). Carboplatin, paclitaxel, and trastuzumab combination therapy was initiated. After four cycles, partial clinical response was achieved and a right mastectomy was performed. Pathological results indicated multifocal infiltrating ductal carcinoma and were consistent with the previous biopsy.

In September 2009, one month after surgery, recurrence of carcinoma was identified both at the base of the mastectomy and in contralateral lymph nodes, but no distant metastases were found in extensive studies.

First‐line treatment for advanced and trastuzumab‐resistant disease using capecitabine (1000 mg/m^2^ PO BID on days 1‐14) plus lapatinib (1250 mg PO on days 1‐21) every three weeks was proposed. After six cycles, lesion in left lymph node and skin lesions on the base of the mastectomy scar disappeared, achieving a complete response. Therefore, treatment was continued until present time, May 2019, for a total of 144 cycles of treatment. The patient achieved excellent tolerance, except a grade II hand‐foot syndrome at various occasions that required a capecitabine dose reduction and a delay in its administration (every 28 days). After approximately 10 years of treatment, no evidence of regional or systemic disease has been observed and the patient has a good quality of life.

Compared with capecitabine alone, lapatinib in combination with capecitabine increases time to progression (TTP) in patients who have progressed after/on trastuzumab, anthracyclines or taxanes. In one study, the median TTP was eight months in the combination arm and four months in the capecitabine arm (HR 0.49; *P* < .001). However, the evidence supporting the efficacy of lapatinib or its combinations in IBC is scarce.^2^

Lapatinib has shown remarkable activity in IBC in two phase II trials. The response rates are varied, which range from 39% to no complete response, in one study, and from 47% to a single complete response, in the other. Median progression‐free survival in both studies was similar, 15 and 16 weeks, respectively.^3^, ^4^ Lapatinib and chemotherapy combination has also been studied in neo‐adjuvant setting in IBC. In a phase II trial, treatment‐naive patients received lapatinib for 14 days and then lapatinib with weekly paclitaxel for 12 weeks. In HER2 positive patients, combined clinical response rate (clinically evaluable skin disease and RECIST criteria) was 78% and pathological response rate was 18% (Table 1).^5^

**Table 1:** Clinical trials of lapatinib in inflammatory breast cancer and its comparison with pivotal trial of lapatinib and capecitabine in HER2 positive advanced breast cancer

Journal	Pub	Phase	Setting	N	Stage IIIB‐C	Stage IV	Anthrac	Trast M	Lapatinib dose	Combination treatment	ORR	CBR	CR	PR	SD	PFS	OS
NEJM	2006	III	R/R LA or M	163	4%	96%	97%	96%	1250 mg daily	Capecitabine 2000 mg/m^2^	22%	27%	1%	21%	5%	8,4 m	x
Lancet Oncol	2009	II	R/R LA or M	126	22%	78%	84%	75%	1500 mg daily	No	39%	65%	0%	39%	26%	14,6 w	11,2 m
Breast Cancer Res Treat	2013	II	R/R LA or M														
				Coh 1 (38)	16%	84%	83%	55%	1500 mg daily	No	29%	66%	3%	26%	37%	16,1 w	14,7 m
				Coh 1 (38)	16%	84%	83%	58%	1500 mg daily	Pazopanib 800 mg daily	45%	61%	11%	34%	16%	14,3 w	16,2 m
				Coh 2 (36)	19%	75%	85%	50%	1500 mg daily	No	47%	80%	3%	44%	33%	16 w	15,9 m
				Coh 2 (38)	34%	63%	85%	50%	1000 mg daily	Pazopanib 400 mg daily	58%	84%	0%	58%	26%	16 w	x
J Clin Oncol	2010	II	Neoadjuvant														
				Coh A (42)	83%	17%	0%	0%	1500 mg daily	Weekly paclitaxel	79%	86%	0%	79%	7%		x

Note

Journal, abbreviated title of journal; Pub year, year of publication of the article; Phase, clinical trial phase; Setting, R/R LA or M (relapsed/refractory locally advanced or metastatic breast cancer); N, number of patients included (Coh: Cohort [only cohorts containing monotherapy or combination lapatinib treatment have been included]); Anthrac, previous treatment with anthracyclines; Trast M, previous treatment with trastuzumab for metastatic disease; ORR, objective response rate (complete response rate and partial response rate: combining of clinically evaluable skin disease criteria and RECIST criteria, if applicable); CBR, clinical benefit rate (ORR and stable disease rate); CR, complete response rate; PR, partial response rate; SD, stable disease rate; PFS, progression‐free survival; OS, overall survival; W, weeks; M, months; X, data not available.

Lapatinib and capecitabine for IBC were used in two case reports. In one of the case reports, a heavily pretreated patient had a local progression after 6 months of receiving 1250 mg per day of lapatinib and 1000 mg of capecitabine per square meter days 1‐14. Metronomic capecitabine was then started, 500 mg thrice daily, with the same dose of lapatinib, and disease control was achieved for 20 additional weeks.^6^ In another case, double blockade with lapatinib and trastuzumab has been reported to be as effective as maintenance therapy in stage IV IBC, after initial treatment with chemotherapy and trastuzumab combination. The patient achieved stable metastatic disease during four years, and she is still in treatment.^7^

Some studies have addressed the issue of long‐term survival with anti‐HER2 therapy, although most were based on trastuzumab (Table 2), not lapatinib, and none was specifically directed at IBC.

**Table 2:** Factors associated with long‐term survival with trastuzumab anti‐HER2 therapy for metastatic disease

First author (Journal)	Pub year	Long‐term survival‐associated factors
Gamez‐Pozo A (PLoS One)	2014	No trastuzumab as adjuvant therapy
		No alterations in PI3K‐mTOR pathway
Yardley DA (Br J Cancer)	2014	HR positivity
		Mtx to bone or bone + breast or node/local sites
		First‐line trastuzumab plus taxane use
Harano K (Breast Cancer Res Treat)	2016	HR positivity
		Low burden of disease
		Mtx to bone and soft tissues
		Resection of the mtx site and primary tumour
Murthy P (Breast Cancer Res Treat)	2016	Younger age at diagnosis
		Lower stage
		HR positivity
		Only one organ involved at diagnosis
Omarini C (Cancer Biol Ther)	2018	No central nervous system spread
		Low burden of disease
		No trastuzumab as adjuvant therapy

Note

Journal, abbreviated title of journal; Pub year, year of publication of the article; HR, hormone receptor; Mtx, metastasis.

About toxicity, our patient had limitant dose toxicity. In some studies, lapatinib‐associated mucocutaneous toxicities have been proposed as predictors of improved progression‐free survival in HER2‐positive IBC patients, but we do not know the repercussion of this aspect yet.^8^

Some difficulties stand out in the management of IBC, particularly the definition of it which is usually based on clinical criteria, dermal lymphatic invasion is not mandatory, as well as in defining response, since a clinical response may or may not be accompanied by a radiological response at the same time.^9^

The improvement of response and survival outcomes in IBC involves investigating new pathways and molecular targets, as well as identifying biomarkers that determine which patient will benefit of a drug already studied like lapatinib.^10^
